# Successful approach for emergent consent for ECMO research

**DOI:** 10.1186/cc14359

**Published:** 2015-03-16

**Authors:** J Board, S Vallance, C Aubron, D Pilcher, V Pellegrino, DJ Cooper

**Affiliations:** 1 The Alfred Hospital, Melbourne, Australia; 2 Monash University, Melbourne, Australia

## Introduction

The HELP-ECMO pilot study (Heparin low dose protocol versus standard care in critically ill patients undergoing ECMO; ACTRN12613001324707) is a randomised controlled phase II study evaluating two levels of heparin anticoagulation in patients with no requirement for full anticoagulation. This work is a substudy of the HELP-ECMO trial and describes the consent process of the parent study. At our site, consent for research is often obtained by the research coordinator with little involvement from investigators. However, the nature of the ECMO population required a modified consent approach to be implemented given that ECMO is often inserted emergently. It required a model that would be successful out of hours and could be delivered by any member of the treating team.

## Methods

The HELP-ECMO pilot study is enrolling patients admitted to a large metropolitan ICU who require ECMO. Education on eligibility criteria and study processes was given to all ICU senior medical staff. Consent must be performed prior to the commencement of anticoagulation, often only a short time after ECMO cannulation. To facilitate recruitment at all hours, a HELP-ECMO study box is located in the ICU. Within the box is an instruction page outlining the screening, consent and randomisation process, as well as administrative tasks. Plain language statements, randomisation envelopes and consent documentation stickers are provided. A consent script is provided to ensure consistency across consenting personnel. The process is reviewed by the research coordinator the following day to confirm local governance compliance.

## Results

From April to December 2014, 30 patients were screened. Fourteen met the eligibility criteria and were approached for consent. Twelve patients were enrolled and randomised to receive either standard anticoagulation or low-dose heparin as per the study protocol. Consent was provided by the person responsible for 10 patients. One patient was competent to consent for themselves and one was enrolled under legislation allowing enrolment into research in the absence of a person responsible. There were two refusals. Seventy-one per cent of participants were approached out of hours. Eighty-six per cent were consented by clinicians. Twenty-one per cent of patients were consented by a non-investigator.

## Conclusion

The model of consent described has proven to be successful in this challenging patient population. The ability of all staff to perform consent for the study has been a significant factor in the success of the pilot. The review of study processes by research coordinators has supported this model.

